# Development of ready-to-use tranexamic acid gauze as a hemostatic material for oral surgery: fabrication, hemostasis, and shelf-life

**DOI:** 10.1186/s12903-026-08136-6

**Published:** 2026-03-19

**Authors:** Taechana Sitthikornvanich, Worawut Kriangkrai, Ariya Chantaramanee, Rudjit Tunthasen, Thanyaphat Engboonmeskul, Lalitkorn Promma, Teerawat Sukpaita

**Affiliations:** 1 Department of Oral Surgery, Faculty of Dentistry, Naresuan University, Phitsanulok, 65000 Thailand; 2 Department of Pharmaceutical Technology, Faculty of Pharmaceutical Sciences, Naresuan University, Phitsanulok, 65000 Thailand; 3 Department of Preventive Dentistry, Faculty of Dentistry, Naresuan University, Phitsanulok, 65000 Thailand

**Keywords:** Tranexamic acid, Hemostatic gauze, Oral surgery, Freeze-drying, Fibrin stabilization

## Abstract

**Background:**

Postoperative hemorrhage is one of the major complications of oral surgery. Conventional hemostatic methods, such as chair-side preparation of tranexamic acid (TXA)-soaked gauze or TXA mouthwash, often suffer from dosage variability and increased surgical time. This study aimed to develop a ready-to-use TXA gauze and to evaluate its fabrication process, hemostatic efficiency, and shelf-life stability.

**Methods:**

Gauze samples containing TXA concentrations of 2.5% and 5% (w/v) were fabricated using a freeze-drying technique. Characterization was performed using field emission scanning electron microscope (FESEM) and fourier transform infrared spectroscopy (FTIR). The in vitro blood clotting time (BCT) and plasma recalcification time (PRT) were assessed using independent biological replicates from six healthy volunteers (*n* = 6). Biocompatibility was assessed via MTT assay on 3T3 fibroblasts. Shelf-life stability was monitored over 180 days under International Conference on Harmonisation (ICH) Zone IV conditions (30 °C/65% RH and 4 °C).

**Results:**

The morphological and FTIR measurements confirmed that the gauze fibers were effectively coated with TXA. Meanwhile, the TXA impregnation did not significantly impact the gauze’s physical and biological properties. A burst release of the TXA was observed for the first 3 min, ensuring rapid clot stabilization. In vitro coagulation tests showed that the TXA gauze significantly reduced coagulation times, enhancing the hemostatic process. Similar to 5% TXA gauze, 2.5% TXA gauze had biocompatibility and hemostatic effectiveness. This finding shows that a lower dose (2.5% w/v) achieved hemostasis, maximizing cost-effectiveness and lowering drug toxicity. Stability tests over six months at various temperatures confirmed that the TXA gauze maintained its hemostatic efficacy without significant drug degradation.

**Conclusions:**

The prefabricated TXA gauze is a promising material, especially for patients on anticoagulant therapy, with long-term stability and practical deployment potential in clinical settings.

**Supplementary Information:**

The online version contains supplementary material available at 10.1186/s12903-026-08136-6.

## Introduction

Effective hemostasis is a critical part of oral surgery procedures, particularly following tooth extraction, impacted tooth removal and dental implant surgery [1, 2]. The standard technique employed to stop bleeding from an oral surgery site is to apply pressure using gauze [3]. Nevertheless, the standard approach may not always be effective in preventing the post-operative hemorrhaging. This concern grows more serious in patients with systemic conditions or receiving anticoagulant therapy [1, 4–6]. The development and application of advanced local hemostatic agents have become a focal point in oral surgery research and clinical practice [7].

Traditional hemostatic materials commonly used in oral surgery include: gelatin-based products (Gelfoam^®^, Pfizer, USA; Surgifoam^®^/Spongostan^®^, Ethicon, USA), oxidized regenerated cellulose (Surgicel^®^, Ethicon, USA), collagen-based products (Colla-Plug^®^, Zimmer, USA) [1, 8–10]. These materials are applied to the bleeding space and held in place until hemostasis is achieved, subsequently allowed to dissolve without requiring removal. Numerous studies indicate that the retention of these materials within the wound may yield adverse effects, including allergic reactions, increased inflammation in adjacent tissue, delayed wound healing, potential for abscess formation, interference with bone regeneration, and increased postoperative pain [11–13]. Tranexamic acid (TXA), a synthetic lysine derivative, has emerged as a pivotal antifibrinolytic agent that stabilizes clots by inhibiting plasminogen activation and fibrinolysis [14–16]. Its use is well supported in major and minor surgical procedures, including in patients on anticoagulant therapy [17–20]. Topically applied TXA has demonstrated efficacy in controlling bleeding after dental extractions, with studies reporting significant reductions in both immediate and postoperative hemorrhage events compared to conventional methods [21–24]. In dental practice, TXA is typically administered topically as a mouthwash, an irrigant, or by soaking a standard gauze in a 5% TXA solution (chair-side preparation) prior to application [21, 24–30]. Nonetheless, these application methods exhibit several clinical limitations, including complex preparation requirements, the substantial weight and bulkiness of liquid-based delivery forms, difficulty with transportation, storage difficulties, and a short shelf life [31]. To address these limitations, recent attention has turned to the development of prefabricated TXA hemostatic material, which can be directly applied to the surgical site, offering immediate and sustained antifibrinolytic effects while eliminating the risks associated with patient-led preparation. This is particularly relevant as traditional methods, such as patients dissolving TXA tablets at home for mouthwashes, are prone to dosage inaccuracies and inconsistent preparation, which can compromise therapeutic outcomes. This study aims to evaluate the fabrication, in vitro hemostatic performance, and shelf-life stability of a prefabricated TXA-impregnated gauze.

## Materials and methods

### Fabrication of the TXA gauze

The medical cotton gauze fabrics (5 × 5 cm², made from 100% cotton) were purchased from Udomphan Supply Co., Ltd. (Nakhon Pathom, Thailand). In the preparation of prefabricated TXA-impregnated gauze, TXA solution 25 mg/ml or 50 mg/ml (OLIC (Thailand) Limited, Ayutthaya, Thailand) in a volume of 2.5 ml was poured on a cotton gauze fabric in a 60 mm plastic Petri dish (Bioscan Inc, China). This resulted in a theoretical drug loading of 62.5 mg for the 2.5% TXA gauze and 125 mg for the 5% TXA gauze per 25 cm² piece. The soaked gauzes were kept at 5 °C for 1 h and then frozen at −80 °C overnight. Finally, the soaked gauzes were freeze-dried at −50 °C (Labconco, USA) for 48 h to obtain 2.5% TXA gauze and 5% TXA gauze. The fabrication method of TXA gauze is shown in Figure S1.

### Surface morphology

The pretreatment of pure gauze, 2.5% TXA gauze, and 5% TXA gauze for observation was the process of gold spray (SPI Supplies, USA). Morphology of the gauzes was observed and recorded under a Field Emission Scanning Electron Microscope (FESEM) (Thermo Fisher Scientific Apreo S, USA) operating at an accelerating voltage of 10 kV and a working distance of approximately 10.3 mm.

### Fourier transform infrared spectroscopy

The chemical composition of pure gauze, TXA, and both groups of prepared TXA gauze was investigated by attenuated total reflectance/Fourier transform infrared spectroscopy (ATR/FTIR) on the FTIR spectroscopy (Perkin elmer, USA) at room temperature in the standard frequency range (4000–400 cm^− 1^).

### Mechanical testing

The tensile strength of both the wet and dry states of pure gauze and prepared TXA gauze was determined using a universal testing machine (Instron, Japan) with a velocity of 2 mm/min following ASTM D882-12 guidelines. The gauzes were cut into a rectangular shape with a gauge length of 40 mm and a width of 20 mm. Young’s modulus was calculated from the linear slope of the stress-strain curve to ensure consistency in reporting. All measurements were performed in triplicate (*n* = 3).

### Blood collection

This study was approved by the Research Ethics Committees of Naresuan University (Approval No: 059/2025). The study protocol confirmed that written informed consent was obtained from all participants. To account for inter-individual variability in coagulation profiles, venous blood samples were collected from six healthy volunteers (*n* = 6). Informed consent was obtained from 6 volunteers before the use of their venous blood for evaluations following protocol approved as per Helsinki guidelines. Healthy subjects with an age range between 18 and 65 years were included if none of the following exclusion criteria were met: recent intake of medications that could interfere with the results of the study (such as anticoagulants or antiplatelet medications, nonsteroidal anti-inflammatory drugs such as ibuprofen, acetylsalicylic acid, or selective serotonin reuptake inhibitors) 10 days prior blood collection, pregnant or breastfeeding women, or the presence of hereditary or acquired coagulation disorders.

The 10 ml of blood from each subject was drawn by clean venipuncture from an antecubital vein using a 21-gauge butterfly cannula system (Nipro Co. Ltd, Thailand). Blood was collected into plastic syringes (Nipro Co. Ltd, Thailand) and used immediately for the further experiments.

### Water and whole blood absorption

Each gauze sample was weighed (W_0_) before tests after being submerged in 10 mL of phosphate-buffered saline (PBS, pH 7.4) or citrated whole blood for 5 min at 37 °C. Subsequently, the gauze samples were taken out from the solution and were placed on a glass plate which formed an angle of 45° from level for 1 min to remove the redundant PBS and weighed for a second time (W_1_). The fluid absorption (%) of the sample was measured by the following equation:

$$The\;fluid\;absorption\;(\%)\;=\;((W_{1}\;-\;W_{0})/W_{0})\;\times\;100\;\;$$

### Drug release study

The TXA release study was conducted using the full-sized prefabricated gauze (5 × 5 cm²) to represent its actual clinical application. The gauze was immersed in 30 mL of PBS (pH 7.4) and maintained at 37 ± 0.5 °C. At specific time intervals (1, 3, 5, 10, 15, 30, and 60 min), 1 mL of the medium was collected and replaced with an equal volume of fresh PBS. The concentration of released TXA was quantified using a UV-visible spectrophotometer (Thermo Fisher Scientific, USA). While various wavelengths have been suggested in previous studies, our preliminary method optimization conducted by scanning the absorption peaks of TXA at multiple concentrations identified 220 nm as the most reliable and sensitive wavelength for this study. The analytical method was validated for linearity, and the concentration of the released TXA was back-calculated using the regression equation obtained from the established standard curve (Y = 0.1676X + 0.0361, (*R*^2^ = 0.9993). All formulations were analyzed in triplicate (*n* = 3). The cumulative drug release percentage was calculated using the following equation:

$$Cumulative\;release\;(\%)\;=\;(Total\;amount\;of\;drug\;released\;at\;time\;t\;/\;Initial\;drug\;loading)\;\times\;100$$

### Cytotoxicity assay

According to the MTT assay, the viability was measured on the 3T3 cells. The gauze samples and commercial hemostatic gauze product (QuikClot combat gauze, TeleflexTM, NC, USA) were sterilized under UV light for 30 min. For the indirect contact test, a piece of gauze sample (0.2 g/ml) was immersed in a culture medium for 24 h at 37 °C. The supernatant obtained was applied to the monolayer culture of 3T3 cells, incubated for 1 and 3 days, then added 10 µl of MTT per well with a subsequent incubation for 2 h, and measured the absorbance at 540 nm. The cells cultured without a gauze sample were served as a control. The cell viability (%) was calculated by the following equation:

$$Cell\;viability\;(\%)\;=\;OD\;sample\;/\;OD\;control\;x\;100$$

### Hemostatic efficiency assessments

In the hemostatic efficiency tests, QuikClot was utilized as a positive control. Despite its distinct kaolin-based mechanism which activates the intrinsic coagulation pathway and its known cytotoxicity toward oral soft tissues, QuikClot was selected because it represents the only commercially available prefabricated hemostatic gauze. Therefore, QuikClot served as a functional benchmark for evaluating the performance of our newly developed TXA-impregnated gauze.

### Blood clotting time (BCT)

The BCT test is a simple method used to evaluate whole blood coagulation in vitro. Briefly, plain gauze, 2.5% TXA gauze, 5% TXA gauze, and QuikClot were immersed in 30 mL of PBS at 37 °C. 100 µl aliquots were taken at a submersion time of 5 min and put in an Eppendorf tube. Then 250 µl of 3.2% sodium citrated blood was added to the sample, followed by vortexing for 10 s, and incubated for 3 min at 37 °C. After that, 25 µl of 0.25 M calcium chloride (CaCl_2_) (Loba Chemie Pvt. Ltd, India) aqueous solution was pipetted into the tube to activate blood coagulation. The tube was tilted every 15 s until the blood stopped flowing through the wall of the tube. The clotting time was used as the result of the BCT test.

### Plasma recalcification time (PRT)

Plasma recalcification time is the time required for recovery of the intrinsic blood coagulation process after adding Ca^2+^ to the calcium-removed plasma. The plasma calcium recovery test is a simple and direct method to simulate an intrinsic coagulation process *in vitro.* It is mainly used in the evaluation of the ability of platelets to form thrombus, which is important in hemostasis studies. Briefly, blood was collected with anticoagulant sodium citrate (9:1). To obtain the platelet-poor plasma (PPP), centrifugation of collected samples was carried out at 5000 rpm for 12 min. The fresh 250 µl of PPP was incubated with 100 µl aliquots of pure gauze, 2.5% gauze, 5% TXA gauze and QuikClot for 3 min at 37 °C. The recalcification of plasma was done using 25 µl of 0.25 M calcium chloride solution. Plasma was observed every 15 s to estimate silky fibrin thread formation in PPP. PRT was designated as the time to recalcification from the time of addition of CaCl_2_. Each sample was tested three times.

### Fibrin network structure observation

Platelet-rich plasma (PRP) was obtained by centrifugation of 3.2% sodium citrate-treated whole blood at 4 °C at 100×g for 10 min. The plain gauze, 2.5% TXA gauze, 5% TXA gauze and QuikClot were washed thrice with deionized water followed by a rinse with 0.1 M PBS. 500 µl of PRP and 50 µl of 0.25 M calcium chloride solution were dropped onto each sample surface and incubated for 60 min at 37 °C. They were further washed to remove plasma proteins and non-adhered platelets with PBS and reacted with 2% glutaraldehyde solution (BDH Chemicals Ltd, UK) for 1 h. The samples were washed again, dehydrated by treatment with an ethanol gradient (30%, 50%, 70%, 90%, and 100% v/v, RCI Labscan, Thailand), dried in room temperature and stored in a desiccator. Fibrin network structure on the samples was evaluated by FESEM.

### Drug stability and shelf-life of TXA gauze

To evaluate the drug stability and shelf-life of TXA gauze over a 6-month period, 2.5% TXA gauze and 5% TXA gauze in sterilization roll were packed in the opaque zip lock bag, simulating the final intended packaging. The packed samples were immediately stored and randomly allocated into two groups of different storage conditions aligned with the ICH guideline for real-world hot/humid climates [32]: (1) Long-term condition at 30 °C with 65% relative humidity. (2) Refrigerated conditions at 4 °C with 65% relative humidity. The 4 °C condition was specifically included to simulate actual clinical storage environments and to investigate whether lower temperatures affect the drug’s stability or the gauze’s hemostatic bioactivity. The stored samples were collected for subsequent evaluation at 0, 15, 30, 90, and up to 180 days. BCT test was used for evaluation the real-time drug stability and shelf-life of TXA gauze. Figure S4 presents a schematic representation of the storage protocol.

### Statistical analysis

The results are expressed as the mean ± standard deviation (SD). Statistical analysis was performed using one-way ANOVA followed by Tukey’s post-hoc test for multiple comparisons (*n* = 6 biological donors). A p-value of < 0.05 was considered significant.

## Results

### Characterization and physical properties of ready-to-use tranexamic acid gauze

**Fig. 1:**
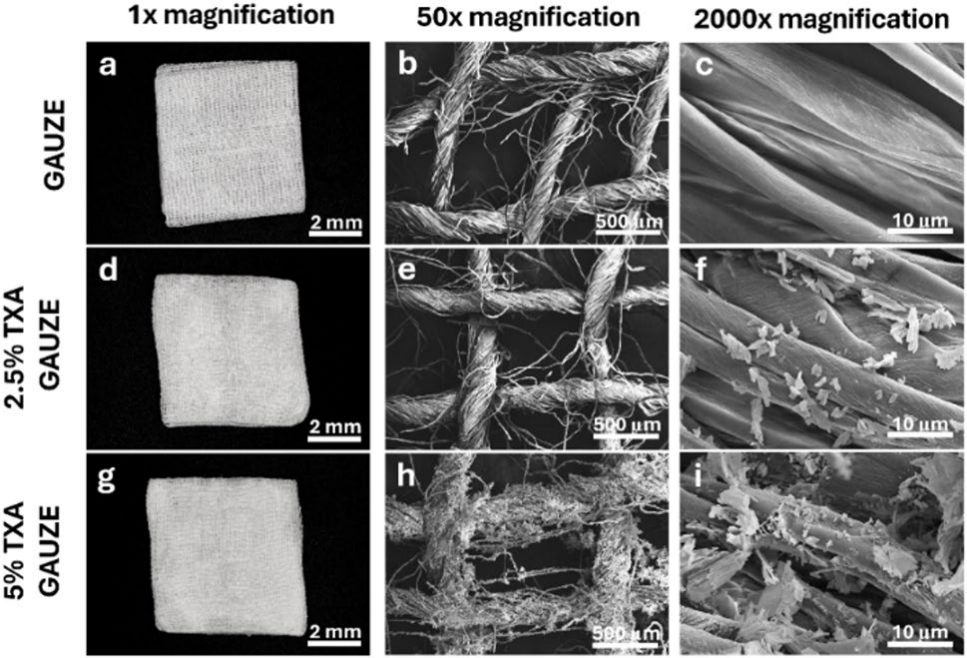
Morphological characterization of hemostatic materials. **a**–**i** Photographs and FESEM micrographs of plain gauze, 2.5% TXA gauze, and 5% TXA gauze at 1x, 50x, and 2000x magnification. Successful impregnation is evidenced by the density of TXA crystals on the fiber surfaces

The ready-to-use tranexamic acid gauze was successfully constructed by a simple freeze-drying technique. The external appearance and FESEM microstructure of bare gauze and both groups of TXA gauze were shown in Fig. 1. Upon examining a standard photograph without magnification, the external shape and morphology of the gauze samples remain unchanged after the medicated and freeze-dried process (Fig. 1a, d and g). However, when examined in high-magnification FESEM images, the microstructure of the gauze fibers altered markedly, with the accumulation of irregularly shaped molecules on their surface (Fig. 1e, f, h and i). These molecules exhibit an increase in density according to the rise in TXA concentration, indicating the successful coating of TXA on the surface of the gauze fibers.

**Fig. 2:**
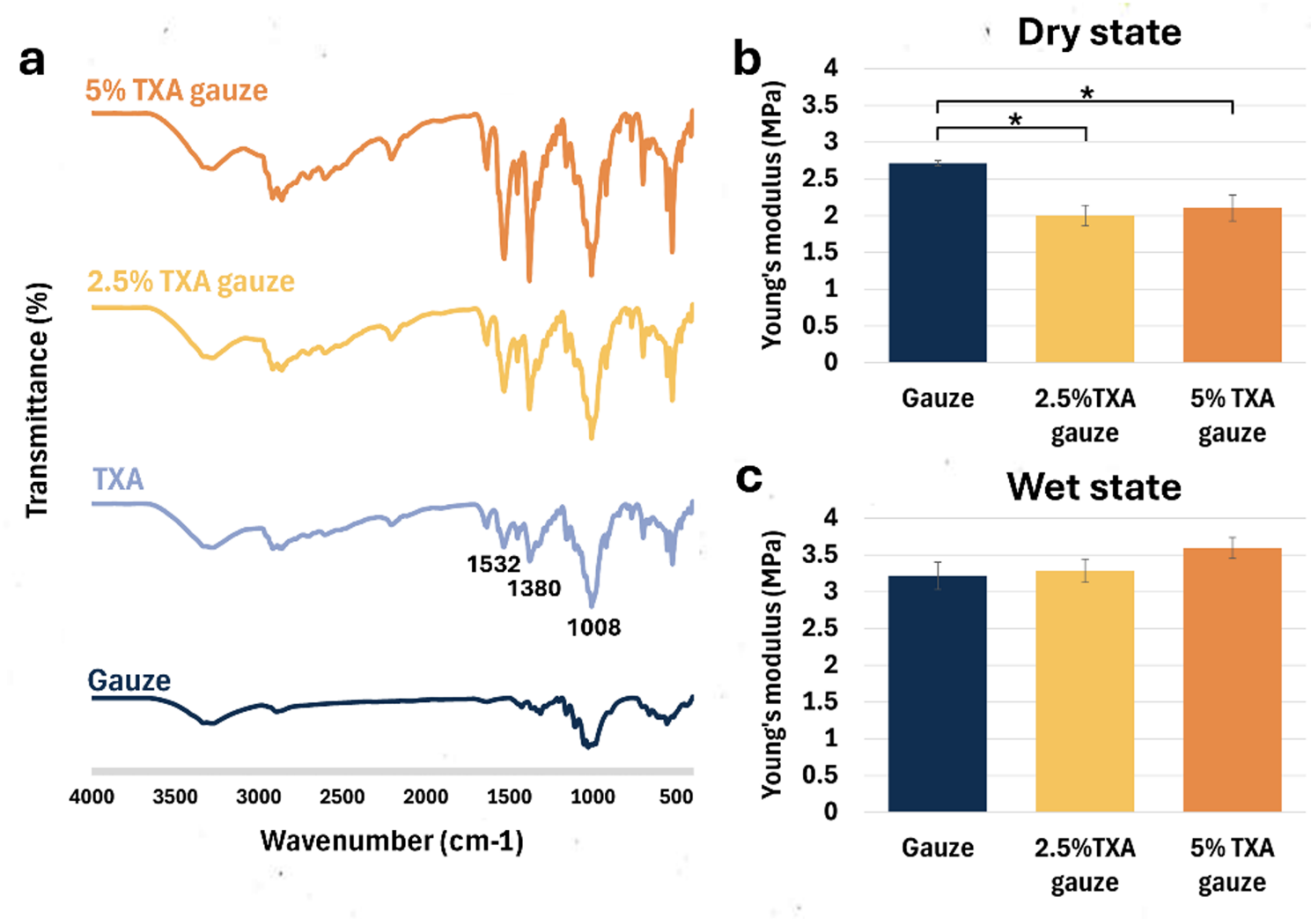
Physical characterization of TXA gauze in various TXA concentrations. **a** The FTIR data from TXA gauze show the characteristic absorption peaks for TXA at 1008, 1380, and 1532 cm^− 1^. **b**, **c** The presence of TXA resulted in a decrease in the tensile strength of the dry state of TXA gauze (*n* = 3, technical triplicate). Values are defined as mean ± SD. *P < 0.05 compared to plain gauze

FTIR spectra of gauze, TXA, 2.5% TXA gauze, and 5% TXA gauze were presented in Fig. 2a. The spectrum of plain gauze exhibited characteristic peaks of cellulose, including a broad band at approximately 3334 cm^− 1^ (-OH stretching), and 2894 cm^− 1^ (C-H stretching). In pure TXA, the N-H stretch of the amide appears at the FTIR absorption peak at 1532 cm^− 1^, while the absorption peaks at 1380 and 1008 cm^− 1^ indicating the presence of the carboxylic functional group and C-N stretching vibration, respectively. Spectrum overlays from TXA gauze (2.5% and 5%) show similar peaks with pure TXA. Furthermore, the graph displayed a more prominent peak correlated with an increase in TXA concentration. FTIR results from TXA gauze exhibit the expected absorption peaks for TXA, indicating that TXA was well incorporated into the gauze fibers without significantly altering the drug’s functional structure.

**Fig. 3:**
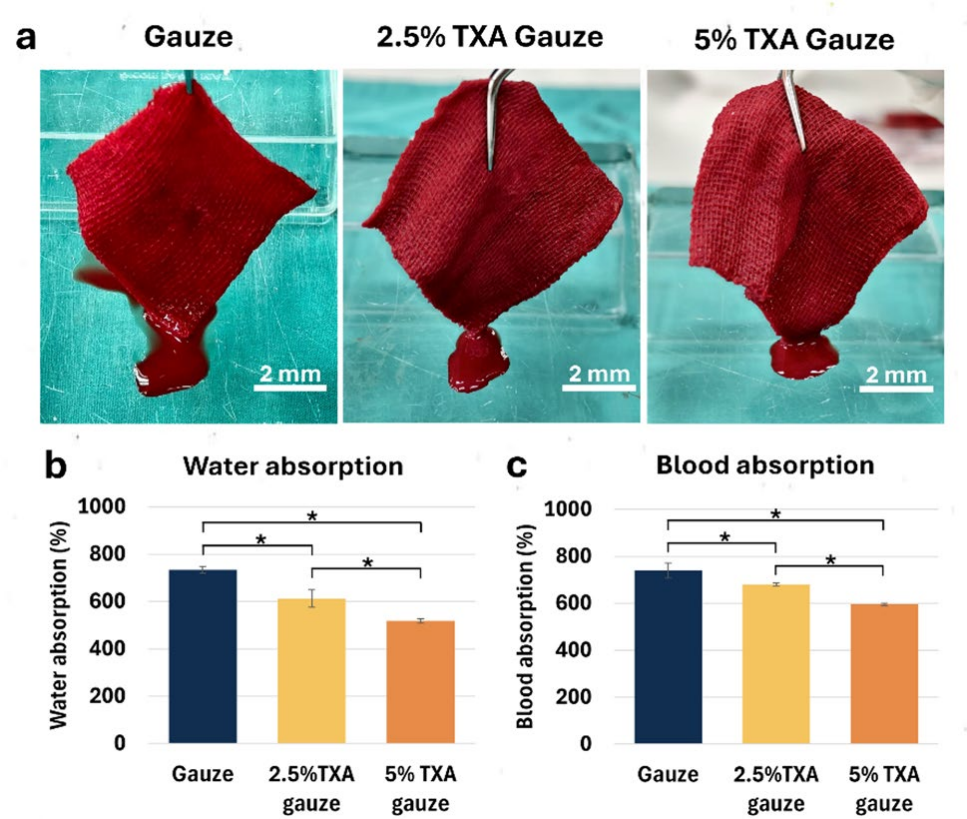
Fluid absorption characteristics. **a** Visual representation of whole blood absorption. **b**, **c** Quantitative water and blood absorption capacity (*n* = 3, technical triplicate). Values are defined as mean ± SD. *P < 0.05

The tensile strength of TXA gauze in both dry and wet states was measured to characterize its mechanical properties. In the dry condition, it reflects the material’s strength during storage prior to application, whereas in the wet condition, it represents the material’s strength when exposed to the patient’s blood or saliva during actual use. The Young’s modulus of plain gauze, 2.5% TXA, and 5% TXA gauze in dry state was 2.71, 2.00, and 2.10 MPa, respectively. Similarly, in the wet state, Young’s modulus values of 3.22, 3.29, and 3.59 MPa for plain gauze, 2.5% TXA, and 5% TXA gauze, respectively (Fig. 2b and c). Test outcomes indicated that prefabricated TXA gauze treated with the freeze-dried process had a markedly reduced tensile strength. Nevertheless, increasing the concentration of TXA did not influence this property. After immersion for 5 min in PBS or whole blood, all sample groups showed excellent clinical absorption ability, as seen by the homogeneous distribution of fluid on the gauzes (Fig. 3a). However, after weighing the saturated gauze to assess its liquid absorption capacity, the plain gauze had the capacity to absorb approximately 750% of its original weight, and both of the TXA gauze groups demonstrated a significant decrease in water and blood absorption abilities according to the rise in TXA concentration.

### Drug release profile and compatibility

**Fig. 4:**
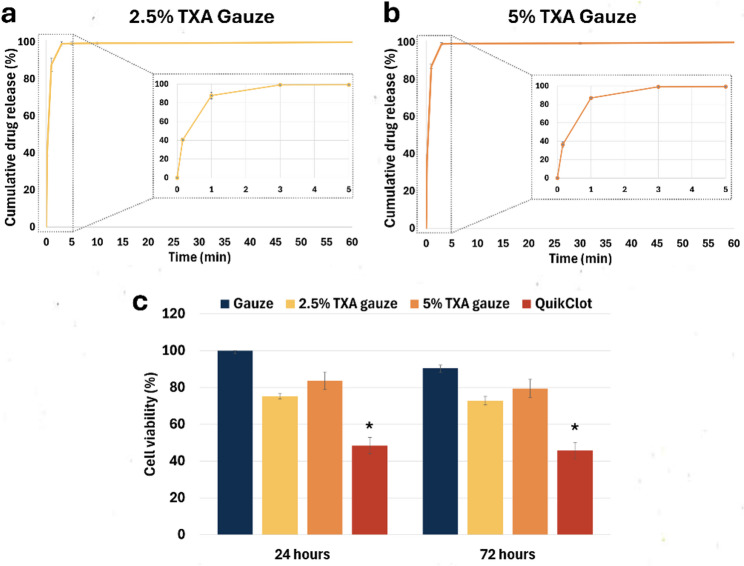
*In vitro* drug release kinetics and biocompatibility. **a**, **b** Cumulative release profiles of 2.5% and 5% TXA gauzes in PBS (pH 7.4) at 37°C. The inset graphs highlight the rapid burst release phase, where approximately 87% of the total drug load was released within the first minute. Quantification was performed via UV-visible spectrophotometry at 220 nm (n=3, technical triplicates). **c** 3T3 fibroblast viability at 24 and 72 hours via MTT assay (n=3, biological replicates). Error bars represent standard deviation (SD). *P < 0.05 compared to the control group

TXA is a highly polar compound with significant water solubility [33]. Therefore, the study of TXA release was undertaken over a short duration of approximately 60 min as shown in Fig. 4a and b. UV–visible spectrometry analysis indicated that the 2.5% TXA and 5% TXA groups had similar TXA release profiles through a diffusion manner. The drug release profiles showed that both 2.5% and 5% TXA gauzes reached 100% cumulative release at the equilibrium state (60 min). This total recovery serves as an indirect quantification of the actual drug content, confirming that the loading efficiency was approximately 100% and aligned with the theoretical doses of 62.5 mg and 125 mg per gauze unit, respectively. A standard curve of TXA at 220 nm is Y = 0.1676X + 0.0361 (R^2^ = 0.9993), where Y and X represent absorbance and TXA concentration (mg/mL), respectively (Fig. S3).

Figure 4a and b shows the drug release of TXA from the gauze patch. A burst release of the TXA was observed for the first 3 min, approximately 87% of the TXA was released rapidly within the first minute, while the remaining 13% was released gradually during the second and third minutes. This is attributable to TXA molecules located on the surface of the gauze fibers and their high solubility.

The MTT colorimetric assay was employed to assess the effect of prefabricated TXA gauzes on 3T3 cells, a family of mouse fibroblast cell lines. The cytotoxicity level was evaluated by comparing four sample groups: plain gauze, 2.5% TXA gauze, 5% TXA gauze, and a commercial hemostasis product (QuikClot combat gauze), as indicated in Fig. 4c. At the 24-hour mark, the cell viabilities (%) for plain gauze, 2.5% TXA gauze, 5% TXA gauze, and QuikClot combat gauze were 100, 75.23, 83.68, and 48.43, respectively. At the 72-hour time point, the graph exhibits comparable characteristics; the plain gauze, 2.5% TXA gauze, 5% TXA gauze, and QuikClot combat gauze showed 90.49, 72.82, 79.42, and 45.73 for the cell viabilities (%), respectively. The results demonstrated that only the commercial group exhibited significant toxicity, whereas the presence of TXA did not induce notable fibroblast cell line toxicity.

### Effect on coagulation cascade

**Fig. 5:**
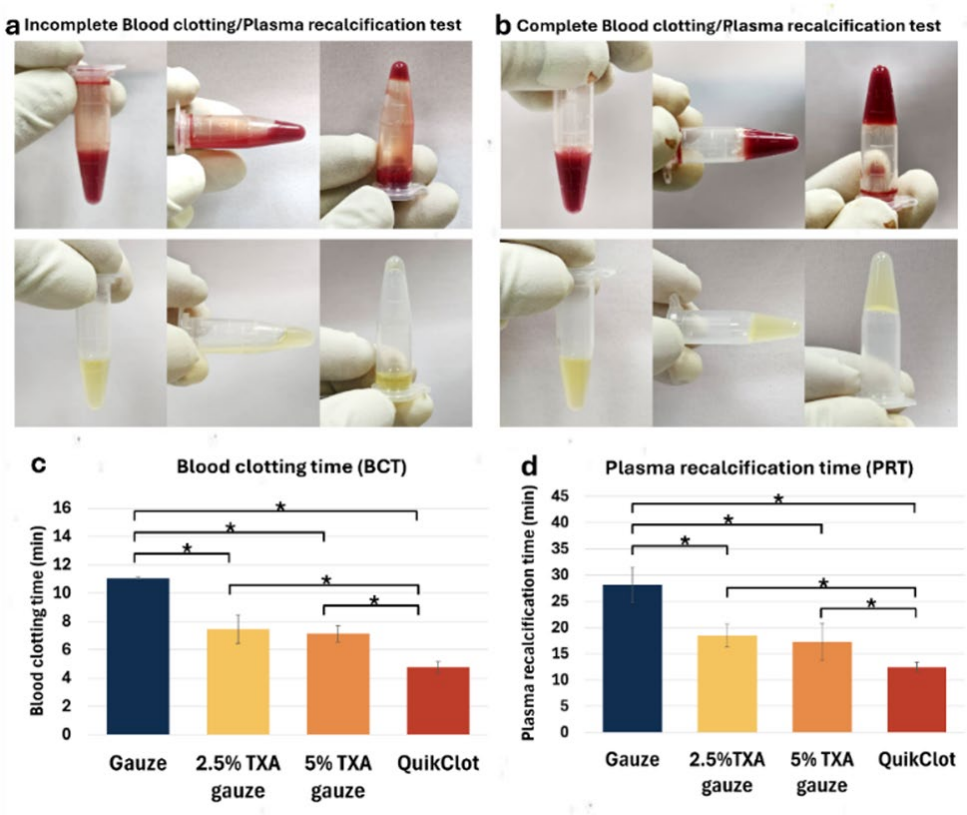
Evaluation of the coagulation cascade. **a**, **b** Representative images of incomplete vs. complete blood clotting and plasma recalcification. **c**, **d** BCT and PRT results (*n* = 6 donors). TXA groups show significantly shorter times than plain gauze (*P < 0.05)

In order to evaluate whether prefabricated TXA gauzes can enhance the coagulation process, BCT and PRT were employed to analyze the overall coagulation cascade and the intrinsic pathway cascade, respectively. Both tests work on a similar principle: a small Eppendorf tube containing blood or PPP was inverted every 15 s and visually examined for flow within the tube. Continued fluid movement indicated incomplete coagulation (Fig. 5a). If fluid was unable to flow, it signifies complete coagulation, and this point was noted as the result of the test (Fig. 5b). Overall, as shown in Fig. 5c and d, the BCT and PRT tests showed identical results. The BCT and PRT of the TXA-coated gauze groups were significantly shorter than that of the plain gauze (*P* < 0.05). The 2.5% and 5% TXA gauze groups exhibited no significant differences in results, whereas the QuikClot combat gauze group demonstrated significantly shorter BCT and PRT compared to the other groups (*P* < 0.05). The findings indicated that prefabricated TXA gauzes could not only enhance the overall coagulation cascade (including intrinsic and extrinsic pathways) but also be specific to the intrinsic pathway of coagulation.

**Fig. 6:**
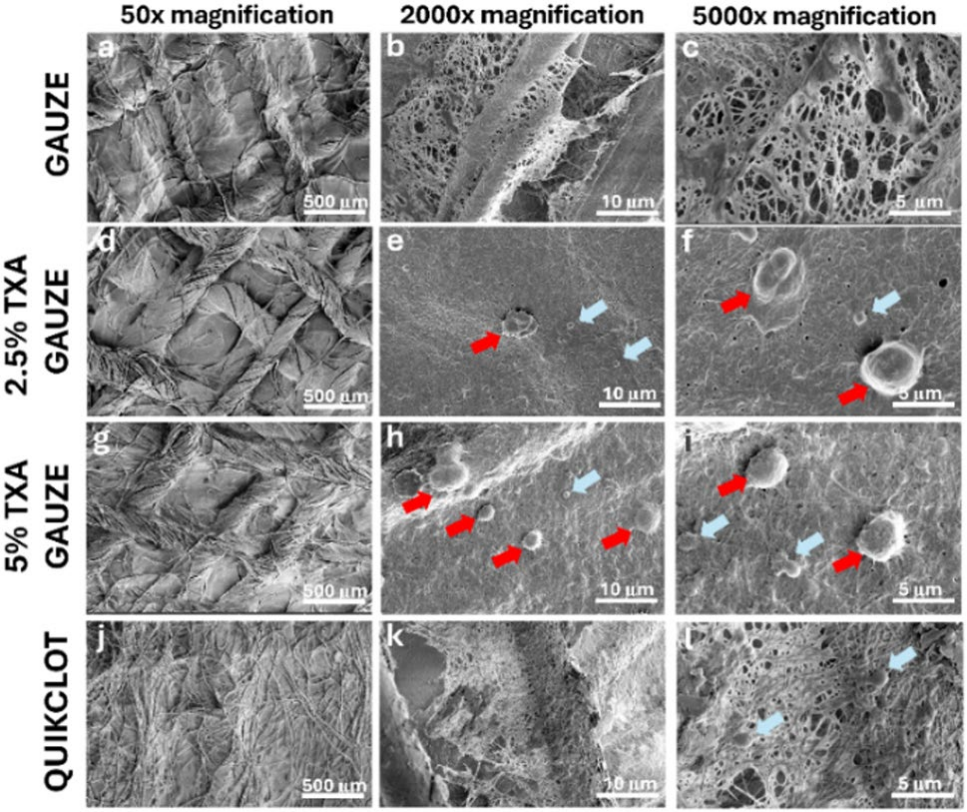
FESEM micrographs of the fibrin network structure. Stabilization of the fibrin matrix on plain gauze (**a**–**c**), 2.5% TXA gauze (d–f), 5% TXA gauze (**g**–**i**), and QuikClot (**j**–**l**) at 50x, 2000x, and 5000x magnification. Red arrows: red blood cells; blue arrows: platelets

Materials were incubated with PRP and allowed to fully coagulate at 37 °C for 1 h, followed by characterization of the fibrin network structure on the material’s surface using FESEM (Fig. 6). The plain gauze controls (Fig. 6a-c) exhibited a loose interconnection of fibrin fibers, and upon examination at the highest resolution, the spaces between the fibers remained clearly observable (Fig. 6c). This difference is clearly seen in both TXA gauze groups (Fig. 6d-i), exhibiting denser fibrin fibers than plain gauze and QuikClot combat gauze positive control. High-magnification images show that the gaps between the fibrils are barely visible, and red blood cells and platelets can be clearly seen embedded in the fibrin framework at the surface of the material (Fig. 6f, i). At low magnification, QuikClot combat gauze exhibits a distinct internal structure compared to other materials, characterized by fibers organized in an irregular, interwoven pattern (Fig. 6j). At higher magnification, the fibrin network resembles that of standard gauze, with a loose pattern and large spaces between the fibrils (Fig. 6k, l). The experimental findings confirmed that the coating of TXA on gauze strongly influenced the stability of the fibrin network structure.

### Real-time drug stability and shelf-life

**Fig. 7:**
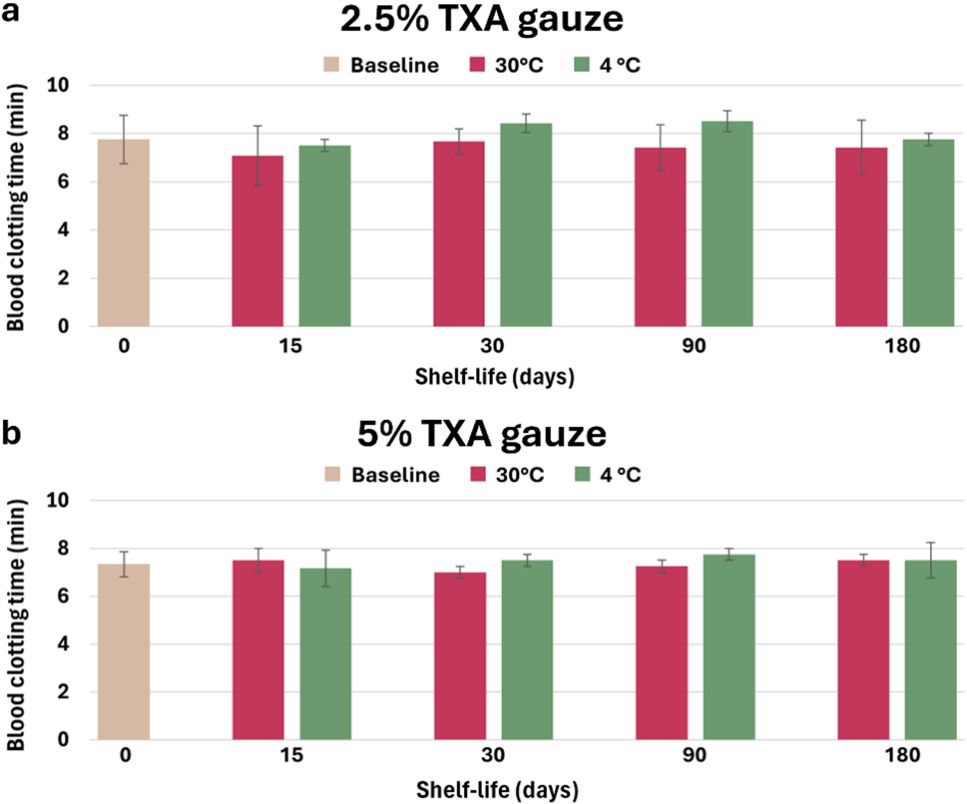
180-day stability and shelf-life assessment. Hemostatic bioactivity (BCT) of 2.5% (**a**) and 5% (**b**) TXA gauze stored at 4 °C and 30 °C (*n* = 6 donors). No significant differences were found compared to the day 0 baseline (*P < 0.05)

Along with good physiological and biological characteristics, the long-term stability of the biomaterial is an important factor for its therapeutic application [34]. The drug stability of TXA gauze was assessed at various storage temperatures over a six-month period, and the bioactivity of the released TXA was examined using the BCT test. Figure S4 displays actual photographs of the sample storage at various temperatures. Figure 7 shows the BCT values derived from testing 2.5% TXA gauze (Fig. 7a) and 5% TXA gauze (Fig. 7b) preserved at various temperatures and for different periods. The BCT values for all experimental groups ranged from 6.25 to 8.75 min, with the mean values of all groups showing no significant difference when compared to the 0-day freshly prepared gauze. The study results confirmed that whether TXA gauzes were stored refrigerated (4 °C with 65% relative humidity) or in a high-ambient environment (30 °C with 65% relative humidity), the TXA released from the gauze preserved the same efficacy in promoting blood coagulation as freshly manufactured TXA gauze. The results indicated that storing the material for six months within a temperature range of 4 °C to 30 °C did not impact the hemostatic properties of TXA gauze.

## Discussion

To reduce the possibility of life-threatening scenarios for patients, the continuation of antiplatelet or anticoagulant therapy during minor dental procedures has been receiving increased attention, highlighting the crucial role of local hemostatic agents used [1, 4, 6, 35–37]. This research aims to develop an economical, simply manufactured, and user-friendly hemostatic material, leading in an innovative prefabricated gauze impregnated with tranexamic acid. TXA was effectively coated onto the gauze fibers using a simple freeze-drying technique, as evidenced by morphological and FTIR measurements.

Although the reduction in tensile strength was noted in dry state samples, the TXA gauze preserved adequate mechanical strength for clinical application. These findings align with a prior study demonstrating that the Young’s modulus of commercial hemostatic dressings, ranging from 0.24 to 0.95 MPa (in the dry state), exhibits acceptable strength and elasticity for wound contact without causing tissue irritation [38]. The ability to absorb liquid is an essential characteristic of medical gauze [39]. In terms of wound dressing, fluid absorption facilitates the formation of a protective barrier. Rapid fluid absorption in compression gauze promotes the collection of blood components at the injury site and accelerates the hemostasis process [40]. The absorption assays revealed that TXA loading reduced the water and blood uptake capacity compared to plain gauze. This can be explained by the following factors: the TXA molecules obstruct the pores between the gauze fibers, the chemical characteristics of TXA alter the hydrophilicity of the gauze fibers’ surface, or the drug may inhibit the swelling or expansion of the gauze fibers upon contact with liquid. These explanations are consistent with studies showing that drug impregnated gauze can modify physical properties and reduced fluid absorption capacity when compared to uncoated gauze [41, 42]. However, the results showed that the water/blood absorption value of the samples ranged from 500% to 800% of their initial weight, aligning with the ideal water absorption range for medical gauze, which is between 100% and 900% of its weight [43], consequently demonstrating that TXA gauze possesses suitable liquid absorption capacity for application as wound closure or compression gauze.

The principles underlying the development of hemostatic materials with drug release abilities differ compared to other drug release materials. In contrast to other materials that necessitate a steady and slowly drug release, hemostatic materials require a rapid drug release that corresponds to the early hemostasis phase, which can occur between seconds to minutes [44]. Besides, the immediate release ensures high local concentration of the drug, improving clot formation and minimizing blood loss effectively [45]. With 87% of the TXA released in the first minute, the TXA gauze showed a rapid release profile, providing immediate hemostatic action at the wound site. This burst release is ideal for procedures requiring rapid clot formation and stabilization. While mechanical friction in the oral cavity is unavoidable, it is not a significant barrier to efficacy. Due to its high water solubility, 87% of the TXA is liberated within the first minute of contact with physiological fluids. This rapid dissolution ensures immediate antifibrinolytic action to stabilize the nascent clot before any mechanical displacement can occur.

The rapid burst release (87% within 1 min) observed in our TXA gauze is strategically designed for common oral surgical procedures, including tooth extractions, third molar surgery, and dental implant placement. In these clinical scenarios, the standard protocol for achieving hemostasis relies on the patient biting on a gauze pack for approximately 30 to 60 min to provide continuous pressure. The immediate release of TXA ensures that a potent antifibrinolytic concentration is delivered directly to the wound site during this initial clotting phase. Theoretically, a hemostatic material capable of sustained release for up to 3 h post-injury would be ideal for enhanced clot stabilization and protection against delayed fibrinolysis [46, 47]. However, the current material may not yet fulfill this requirement as the drug is primarily coated on the fiber surfaces. Developing a second-generation product that combines an initial burst with a sustained-release component over several hours remains a challenge for future investigations.

Various studies have investigated different concentrations of TXA for topical application, frequently between 2.5 mg/ml and 5 mg/ml, with a high of 50 mg/ml [48–50]. Wang et al. [48] showed that topical TXA application for 10 min at a high concentration level (100 mg/ml) does not significantly impact the viability or proliferation of human fibroblasts. In other cell types, including tendon cells or osteoblasts, concentrations over 20 mg/ml may lead to increased cytotoxicity with extended exposure [51]. Biocompatibility testing in this study shown that both 2.5% and 5% TXA gauze significantly outperformed the performance of commercial QuikClot combat gauze in preserving fibroblast cell viability, while being comparable to standard gauze. While the MTT assay results on 3T3 fibroblasts demonstrated excellent biocompatibility for both TXA concentrations, we acknowledge the limitation of using a single non-oral cell line. Given that the material is intended for oral surgical sites, the response of oral-specific cells, such as human gingival fibroblasts or oral keratinocytes, would be highly relevant for further clinical translation. Future studies should incorporate these oral-specific models to more accurately simulate the physiological response of the oral mucosa and further confirm the safety profile of this prefabricated hemostatic gauze. These findings support recent studies indicating that topical TXA treatment enhances hemostasis without adversely affecting wound healing [18, 20, 24].

In the surgical field, TXA can be administered topically using various techniques [52]. The advantage of prefabricated TXA gauze compared to traditional forms is its immediate usability, eliminating the need for complex procedures such as the professional preparation of the appropriate drug concentration. In addition, the freeze-drying procedure for coating the drug onto the gauze is cost-effective and suitable for large-scale production in a factory setting [53]. In vitro hemostatic experiments confirmed the superiority of TXA prefabricated gauze. BCT directly assesses the clotting ability of whole blood, including both cellular and plasma components, whereas PRT assesses plasma factor activity by excluding blood cells and platelets [54]. The significant reduction in both BCT and PRT demonstrated the antifibrinolytic effect of TXA in stabilizing the coagulation cascade. Surprisingly, there was no difference in the effectiveness of the 2.5% and 5% forms. This implies that lower concentrations might be sufficient to achieve the intended hemostatic effect, maximizing cost-effectiveness and lowering the risk of drug toxicity. FESEM analyses of the fibrin network architecture revealed denser fibrin matrices in TXA gauze samples, which further supported the clot structure’s functional stability. These findings are in line with earlier studies showing that TXA increases fibrin’s resistance to fibrinolysis [20, 46, 52]. QuikClot was utilized as a positive control due to its status as a standardized prefabricated hemostat, despite its distinct kaolin-based mechanism. While our results confirm the efficacy of lyophilized TXA gauze, future in vivo or clinical studies should include a direct comparison with freshly impregnated TXA gauze (chair-side preparation). This benchmark will more precisely validate how the lyophilization process preserves drug bioactivity and enhances delivery compared to current clinical practices, facilitating its transition into routine dental care.

In recent years, several advanced delivery systems for TXA have been proposed. For instance, El Halawany et al. developed an alginate/nano-hydroxyapatite composite aerogel loaded with TXA, demonstrating excellent protection against alveolar osteitis and bone formation enhancement in animal models [55]. Other researchers have explored physically crosslinked trilaminate dressings and scaffolds [56]. While these “high-tech” materials show superior biological properties in controlled settings, their clinical adoption in routine oral surgery is often hindered by high production costs and complex application protocols. In contrast, our approach utilizes medical-grade cotton gauze, which remains the “gold standard” in clinical practice due to its unparalleled cost-effectiveness, familiarity to surgeons, and the ability to provide mechanical compression. By transforming standard gauze into a “ready-to-use” medicated format, we eliminate the dosage variability associated with chair-side preparation and significantly streamline the surgical workflow without the prohibitive costs of synthetic scaffolds.

A notable strength of this study is the demonstration of at least 6-month stability of materials following ICH guideline [32]. TXA gauze maintained its hemostatic performance for at least six months even when stored in zone IV hot/humid climatic condition (30 °C with 65% relative humidity). The stability of TXA gauze enables its transport and storage before use, which are essential characteristics for medical products [34]. This feature is frequently challenging to achieve with traditional TXA mouthwashes or soaking bandages, which typically exhibit limited shelf life and storage problems [31]. Despite the positive findings, it is important to acknowledge some of this work’s limitations. First, the absence of in vivo validation remains a limitation. Future investigations will incorporate a rat tail amputation model to quantitatively assess bleeding time and total blood loss reduction. Subsequently, intraoral wound models in rodents or porcine dental extraction models will be used to simulate the oral environment, including salivary flow and mechanical forces. These steps are essential before proceeding to clinical trials in patients on oral anticoagulants to fully validate the material’s potential in dental surgery. Second, the sterilization process is crucial, as it alters the chemical and physical properties of dressing gauze [57]. Sterilization methods such as gamma irradiation, ethylene oxide gas, or autoclaving could impair the physical structure of the gauze fiber and the chemical integrity of TXA. The procedure of ensuring the sterility of TXA gauzes requires further examination. Third, the 180-day stability data provide a promising preliminary assessment of the TXA gauze’s shelf-life under both refrigerated and Zone IV conditions. However, it is important to note that these findings represent initial laboratory-scale observations. For future commercial production and official medical device registration, comprehensive stability studies conducted by certified organizations under a broader range of regulatory conditions remain necessary to fully validate the product’s safety and efficacy for the global market. Finally, in order to facilitate translation into clinical practice, cost-effectiveness, long-term shelf life, and the viability of large-scale manufacturing must be assessed.

## Conclusion

In this work, we developed a ready-to-use tranexamic acid gauze sample by a straightforward freeze-drying process. The gauze used for testing was divided into two drug concentrations: 2.5% TXA and 5% TXA, based on the concentrations commonly used for topical TXA applications. The prefabricated TXA gauze developed in this study represents a simple, scalable, and clinically practical hemostatic material. The gauze sample containing 2.5% TXA exhibited a physicochemical structure, biocompatibility, and hemostatic efficiency comparable to that of 5% TXA gauze. This suggests that a lower concentration was sufficient for achieving hemostasis, maximizing cost-effectiveness, and reducing drug toxicity. Its ready-to-use design overcomes significant drawbacks of existing TXA application techniques and has the potential to greatly enhance perioperative bleeding control, especially for patients undergoing anticoagulant or antiplatelet therapy. Importantly, the material preserved its hemostatic efficacy and stability even after six months of storage under both refrigerated and elevated temperature conditions, addressing practical challenges related to transportation and long-term storage. To verify its effectiveness, optimize dosage, and set standards for its regular use in oral and maxillofacial surgery, additional animal study, clinical trial, and sterilization process optimization are needed.

## Supplementary Information

Supplementary Material 1.

## Data Availability

All data generated or analysed during this study are included in this published article and its supplementary information files.

## Abbreviations

TXA: Tranexamic acid
FESEM: Field Emission Scanning Electron Microscope
ATR/FTIR: Attenuated total reflectance/Fourier transform infrared spectroscopy
PBS: Phosphate-buffered saline
UV: Ultraviolet
MTT: 3-(4,5-dimethylthiazol-2-yl)-2,5-diphenyltetrazolium bromide
BCT: Blood clotting time
CaCl₂: Calcium chloride
PRT: Plasma recalcification time
PPP: Platelet-poor plasma
PRP: Platelet-rich plasma
