# Supplementary material for: Development of ready-to-use tranexamic acid gauze as a hemostatic material for oral surgery: fabrication, hemostasis, and shelf-life
Source: BMC Oral Health. 2026 Mar 19;26:752. doi: 10.1186/s12903-026-08136-6 (PMC13123210; doi:10.1186/s12903-026-08136-6)
Supplement: Supplementary file 1 — Supplementary Material 1. [file 12903_2026_8136_MOESM1_ESM.pdf]

Development of Ready-to-use Tranexamic Acid Gauze as a Hemostatic Material for Oral Surgery: Fabrication, Hemostasis, and Shelf-life

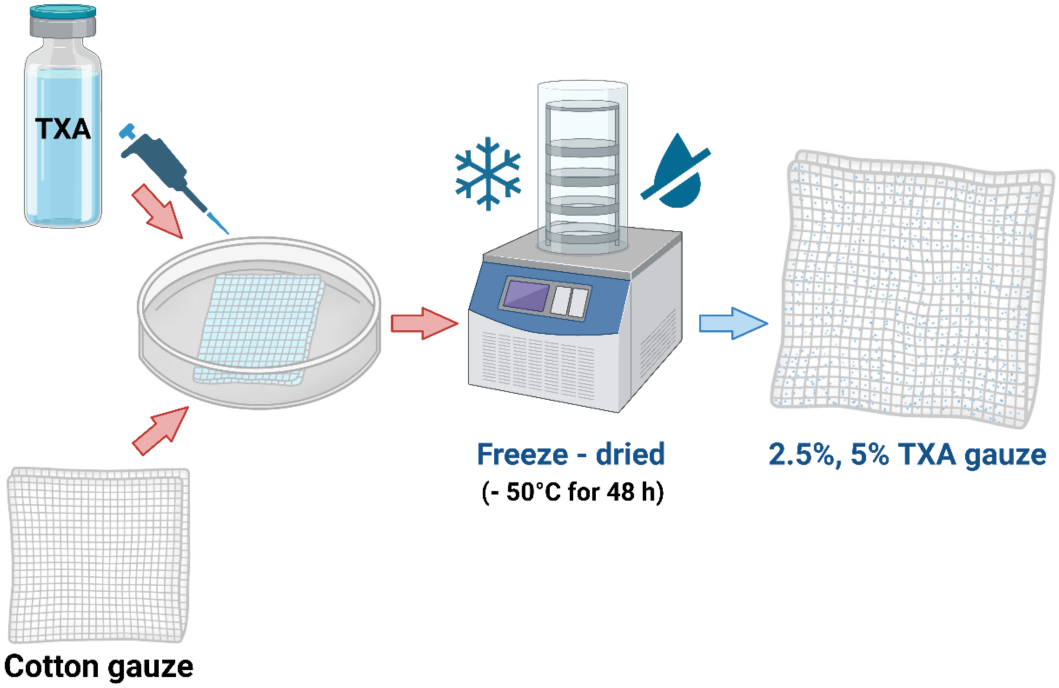

Fig. S1. The fabrication method of TXA gauze. TXA solution (25 mg/ml or 50 mg/ml) in 2.5 ml was dropped onto cotton gauze in a plastic petri dish. The soaked gauzes were frozen overnight and freeze-dried at -50°C for 48 h to obtain prefabricated 2.5% and 5% TXA gauze; figure created in BioRender, S, T. (2025). https://BioRender.com/, (accessed on 17 September 2025).

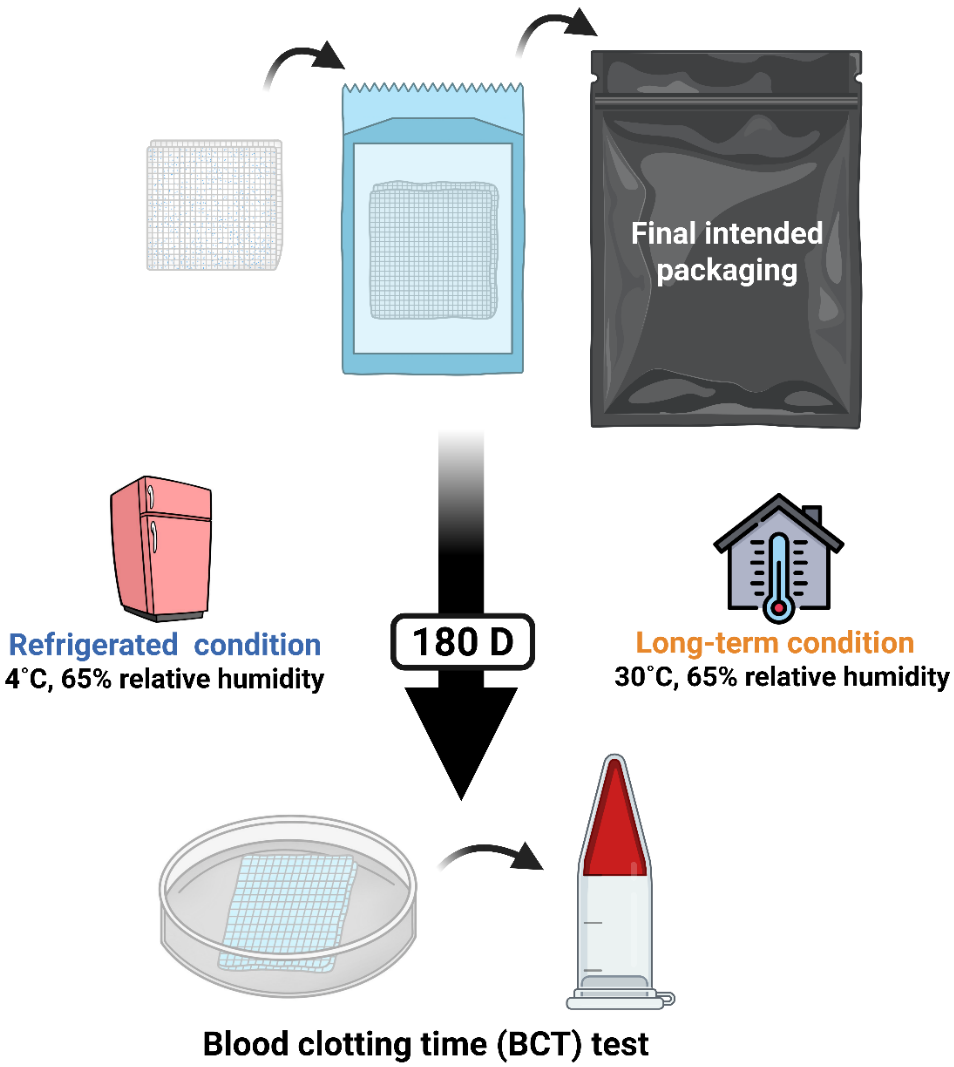

Fig. S2. The shelf-life testing method of TXA gauze. The gauze samples in sterilizing roll were placed in an opaque zip lock container to simulate final packing. The samples were divided into two groups: (1) 30˚C with 65% relative humidity. (2) 4˚C with 65% relative humidity. BCT test were used for evaluation the efficacy of TXA gauze; A figure was created in BioRender, S, T. (2025). https://BioRender.com/, (accessed on 17 September 2025).

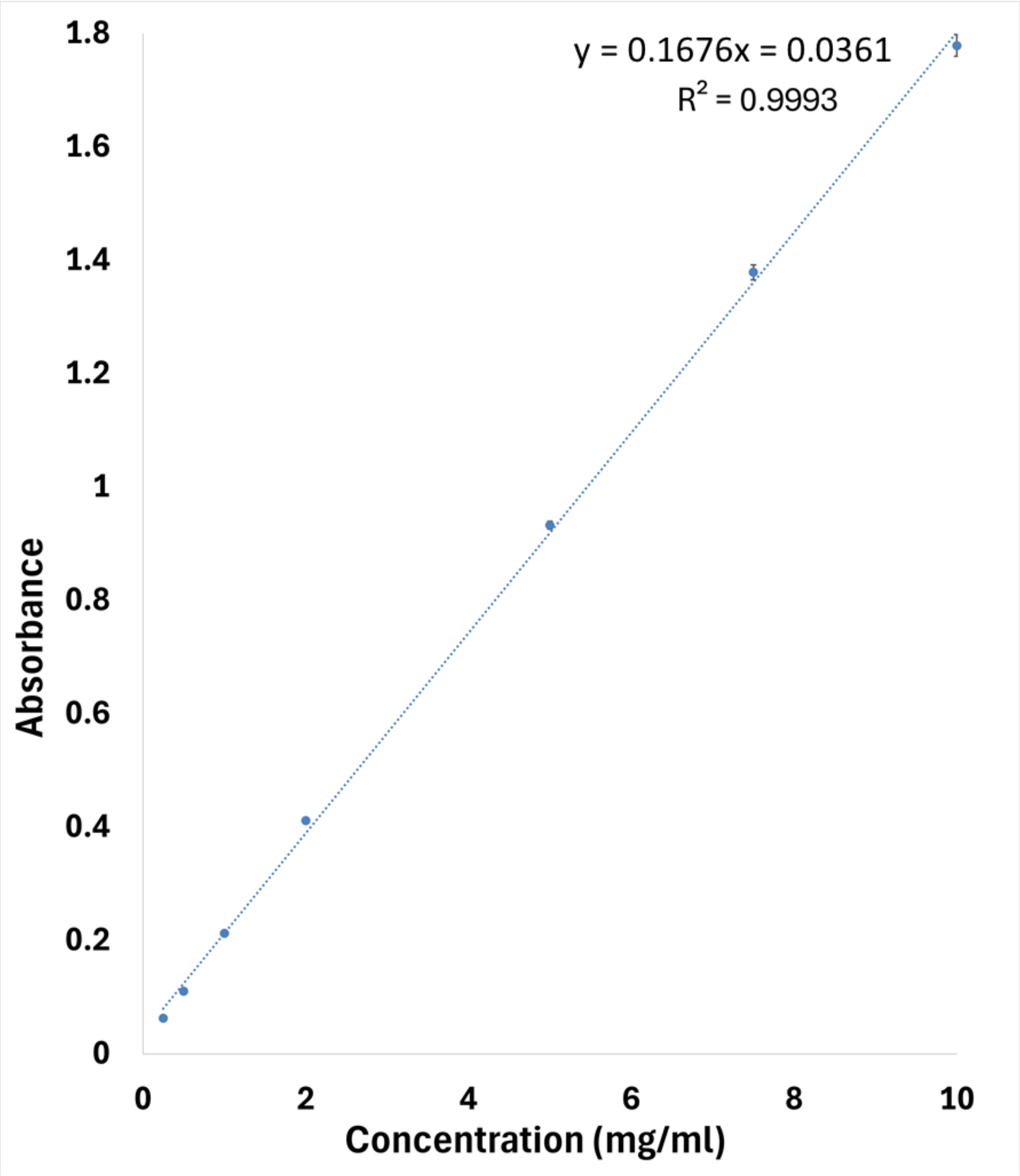

Fig. S3. Standard calibration curve of tranexamic acid (TXA) determined by UV-visible spectrophotometry at 220 nm. Data points represent the mean ± standard deviation (SD) of three independent trials (n=3). The high linearity (R^2 = 0.9993) and minimal error bars reflect the high precision of the analytical method.

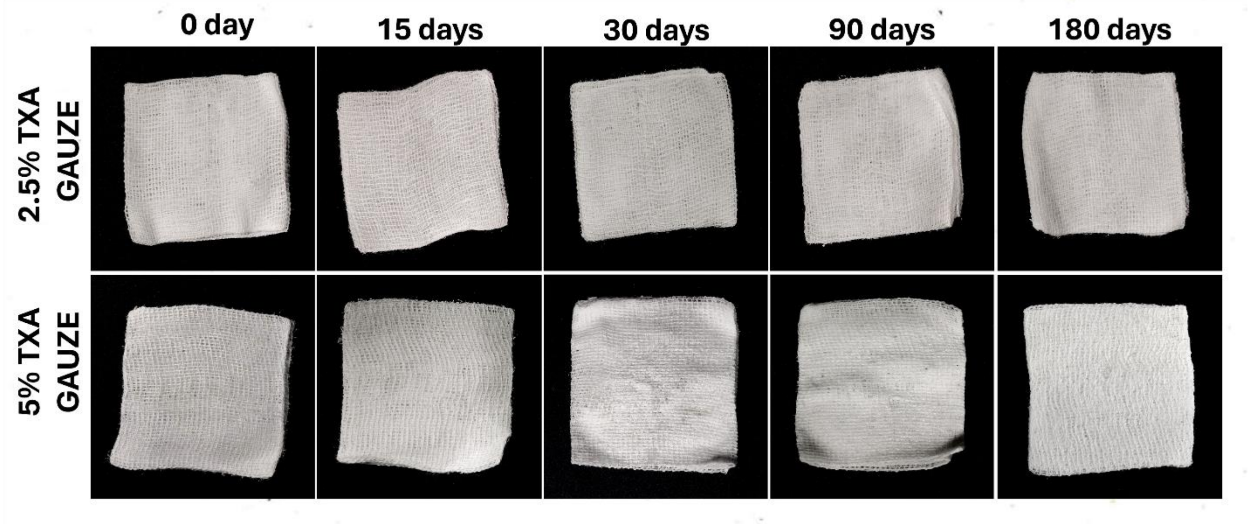

Fig. S4. Actual photographs of the sample storage at various temperatures.

Raw data in each experiment

Table S1: Raw data of tensile strength (dry state)

Young's modulus (MPa)			
Gauze	2.7481	2.6781	2.7033
2.5% TXA gauze	1.8483	2.0303	2.1258
5% TXA gauze	2.1471	2.2556	1.9085

Table S2: Raw data of tensile strength (wet state)

Young's modulus (MPa)			
Gauze	3.4235	3.0676	3.1617
2.5% TXA gauze	3.1609	3.4649	3.2303
5% TXA gauze	3.4304	3.6598	3.6956

Table S3: Raw data of water absorption

Water absorption (mg)		
	W_0	W_1
Gauze	219.3	1863.5
	222.3	1827.2
	217.3	1800.4
2.5%TXA gauze	264.7	1860.3
	260	1895.5
	259.7	1831.5
5%TXA gauze	298.5	1805.4
	289.2	1808.1
	289	1800.7

Table S4: Raw data of blood absorption

Blood absorption (mg)		
	W_0	W_1
Gauze	218.7	1791.5
	213.5	1871.6
	220.2	1813.4
2.5%TXA gauze	255.6	1989.4
	248.5	1928.4
	258.5	2034.2
5%TXA gauze	283.4	1994.6
	286.8	2019.7
	286.1	1989.2

Table S5: Raw data of TXA release of 2.5% TXA gauze

% Cumulative release			
Time	1st	2nd	3rd
10 s	41.39278908	38.36919872	41.63012497
1 min	85.94473183	85.45818843	92.01107213
3 min	99.55661001	97.79688245	99.9762479
5 min	99.52646727	98.53743358	99.8748447
10 min	99.9727743	98.93374759	99.38975371
30 min	99.74816227	99.50659346	99.33402763
60 min	100	100	100

Table S6: Raw data of TXA release of 5% TXA gauze

% Cumulative release			
Time	1st	2nd	3rd
10 s	34.30126808	35.19627804	39.61943679
1 min	85.7548902	87.226784	88.07970658
3 min	99.66932838	98.42422305	99.46419194
5 min	99.11849568	99.29876664	99.56224563
10 min	99.49259751	98.83562451	99.68081266
30 min	98.91209467	99.64286315	99.94707562
60 min	100	100	100

Table S7: Raw data of cytotoxicity

% Cell viability						
	24 hours			72 hours		
Gauze	100	100	100	88.80	90.80	91.90
2.5% TXA gauze	73.60	75.20	76.90	70.10	73.10	75.30
5% TXA gauze	79	84.20	87.80	74	80.20	84.10
QuikClot	44.3	49.20	51.80	41.70	45.60	49.90

Table S8: Raw data of blood clotting time (BCT)

BCT (min)						
Gauze	11	10.75	11.25	11	11	11.25
2.5% TXA gauze	6.25	6.5	7	8	8.5	8.5
5% TXA gauze	6.75	6.75	6.75	7	7.25	8.25
QuikClot	4.25	4.5	4.75	4.75	4.75	5.5

Table S9: Raw data of plasma recalcification time (PRT)

PRT (min)						
Gauze	23.75	25	27.75	29.5	30.75	32.25
2.5% TXA gauze	14.75	18	18	19	20	21
5% TXA gauze	13	13.75	15.75	19.25	20.5	21.25
QuikClot	11.5	11.75	12	12.25	13.5	14

Table S10: Raw data of shelf-life of 2.5% TXA gauze

	BCT (min)					
0 day (Baseline)	6.5	7.25	7.5	7.75	8	9.5
30 ˚C, 15 days	5	6.25	7.25	8	8	8

30 ˚C, 30 days	7	7.5	7.5	7.5	8	8.5
30 ˚C, 90 days	6.75	6.75	6.75	7	8.5	8.75
30 ˚C, 180 days	6.5	6.5	6.75	8.25	8.5	9
4 ˚C, 15 days	7	7.5	7.5	7.5	7.75	7.75
4 ˚C, 30 days	8	8.25	8.25	8.25	8.75	9
4 ˚C, 90 days	8	8.25	8.25	8.5	8.75	9.25
4 ˚C, 180 days	7.25	7.75	7.75	7.75	8	8

Table S11: Raw data of shelf-life of 5% TXA gauze

	BCT (min)					
0 day (Baseline)	6.75	7	7.25	7.25	7.5	8.25
30 ˚C, 15 days	7	7	7.25	7.75	7.75	8.25
30 ˚C, 30 days	6.75	6.75	7	7	7	7.5
30 ˚C, 90 days	7	7	7.25	7.25	7.25	7.75
30 ˚C, 180 days	7.25	7.25	7.5	7.5	7.5	8
4 ˚C, 15 days	6	6.5	7.25	7.5	7.75	8
4 ˚C, 30 days	7.25	7.25	7.5	7.5	7.75	7.75
4 ˚C, 90 days	7.25	7.75	7.75	7.75	8	8
4 ˚C, 180 days	6	7.5	7.75	7.75	8	8
